# How the Age-Friendly Domains Apply to Low-Income Cities and Guide Improvements: Perspectives of Long-Term Residents in New Jersey

**DOI:** 10.1093/poq/nfaf027

**Published:** 2025-08-07

**Authors:** Amanda Jones-Layman, Francine P Cartwright, Seran Schug, Jennifer Kitson, Lisa Siegert, Rachel Pruchno

**Affiliations:** Assistant Professor, School of Social Sciences, Humanities, and Education, Neumann University, Aston PA, US; Project Director, New Jersey Institute for Successful Aging, Rowan-Virtua School of Osteopathic Medicine, Stratford, NJ, US; Assistant Teaching Professor, Department of Sociology and Anthropology, Rowan University, Glassboro, NJ, US; Associate Professor, Departments of Art and Geography, Planning, & Sustainability, Rowan University, Glassboro, NJ, US; Associate Professor, Division of Geriatric Medicine, Cooper University Health Care, Camden, NJ, US; Professor, New Jersey Institute for Successful Aging, Rowan-Virtua School of Osteopathic Medicine, Stratford, NJ, US

## Abstract

The WHO defined age-friendly cities (AFCs) as places with policies, services, settings, and structures that enable people to age in place. Although AFCs have gained attention recently, little is known about the applicability of age-friendly domains to low-income cities. We conducted flexible semistructured interviews with 28 adults aged 65 and older who had lived in New Jersey cities with high poverty rates and low median incomes for at least 15 years.

Participants described their neighborhoods in ways that mapped onto the eight AFC domains. Themes of agency and safety linked the domains. Participants suggested ways to change neighborhoods in low-income cities that would make them age friendly. Findings suggest that the AFC domains have utility as a framework for how older long-term residents of low-income cities describe their neighborhoods. They provide unique information about how these domains relate to one another and identify strategies for making low-income places better environments for older people.

Research consistently finds that aging in place promotes quality of life among older people (Vanleerberghe et al. 2017; Kim et al. 2024). A nationally representative study by the American Association of Retired Persons found that 76 percent of people aged 50 and older want to live in their own homes and communities for as long as possible (Binette 2021). Yet this same study found that just 59 percent of these people anticipate they will be able to stay in their community, either in their current home (46 percent) or in a different home (13 percent). Declining health and homes requiring extensive modifications are well-documented factors influencing whether older people age in place (Oswald et al. 2002; Smetcoren et al. 2017). Less well understood is how a neighborhood’s social, economic, demographic, and physical characteristics impact aging in place, especially as cognitive ability and physical function decline (Beard et al. 2009; Mather and Scommegna 2017; Barnett et al. 2018).

Research using large-scale epidemiological data and multilevel modeling to assess area effects demonstrates the importance of the socioeconomic status of a neighborhood to the well-being of older people (Freedman et al. 2008; Aneshensel et al. 2011; Freedman, Grafova, and Rogowski 2011; Clarke et al. 2012). This research documents the importance of physical characteristics, including greenspaces, sidewalks, safety, the built environment, and access to healthy food. The condition of neighborhood streets and sidewalks can determine mobility for adults who have difficulty walking (Clarke et al. 2008). Accessible neighborhoods are safe and have destinations within walking distance (Satariano et al. 2010; Clarke and Gallagher 2013). Neighborhoods with access to healthy foods have positive effects on older people (Morgenstern et al. 2009; Latham and Clarke 2013; Kaiser et al. 2016). The presence of trash, vandalism, crime, and broken curbs and sidewalks takes a toll on older people (Boardman et al. 2012), while resources such as parks, recreation centers, community centers, and libraries create opportunities for social interaction and physical activity (Clarke et al. 2015).

The ecological model of aging is a key organizing framework for the movement to create age-friendly cities (Menec et al. 2011; Kim et al. 2022). Developed originally by Lawton and Nahemow (1973), the ecological model contended that as personal competence declines, the environment plays a growing role in determining the quality of life. Wahl, Iwarsson, and Oswald (2012) argued that mismatches between personal needs and environmental options undermine well-being in later life.

Large-scale epidemiological studies, however, do not address the complexities underlying the relationship between older people and their neighborhoods. There is evidence, for example, that social inequalities exist between older people who have the resources to make conscious decisions about where and with whom to live and older people who lack such resources (Phillipson 2007). Building on the ecological model, Rowles (1983) suggested that older people who have lived in their community for a long period of time maintain three types of attachment to their environment: *physical insideness* (an intimate familiarity with the physical configuration of the environment), *social insideness* (an integration within the social fabric of the community), and *autobiographical insideness* (reflecting how lifelong accumulation of experiences in a place provides a sense of identity). However, Rowles (1983) focused on a rural Appalachian community, and limited research has examined the experiences of people aging in place in low-income cities (Lewis and Buffel 2020).

The Age-Friendly Cities and Communities Initiative (AFC) was designed to promote active, healthy aging and to help cities plan for an aging population (Plouffe and Kalache 2010). The initiative proposed that an age-friendly city has policies, services, settings, and structures that support and enable people to age in place (WHO 2007). Focus group sessions conducted in 33 cities across 22 countries identified eight interconnected age-friendly domains (https://extranet.who.int/agefriendlyworld/age-friendly-cities-framework/), including outdoor spaces and buildings, housing, respect and social inclusion, social participation, civic participation and employment, transportation, community and health care, and communication and information.

Although the AFC framework has garnered significant attention from social scientists (Torku, Chan, and Yung 2021; van Hoof and Marston 2021; Black and Oh 2022; Greenfield and Buffel 2022), few studies have focused on people living in low-income cities and no studies have examined the experiences of long-term residents of these cities. Yet Jeste et al. (2016) and Buffel, Handler, and Phillipson (2019) argued the importance of developing AFCs in underserved communities. They suggested that strengthening the social fabric of these communities is the primary vehicle for accomplishing this goal. Guided by Rowles’s (1983) conceptual work and the WHO Age-friendly Cities framework, we address the following:

1. How do long-term residents of low-income cities experience their neighborhoods?
2. In what ways do their experiences reflect the AFC domains?
3. What changes relevant to the AFC domains do long-term residents in low-income cities suggest would improve their neighborhoods and make them better places to age?

## Methods

### Sample

Participants included a subset of the Ongoing Research on Aging in New Jersey: Bettering Opportunities for Wellness in Living (ORANJ BOWL) panel. The panel (N = 5,688) was recruited via random-digit-dial beginning in 2006. Panelists were surveyed seven times using quantitative methods between 2006 and 2021. Recruitment criteria included being between the ages of 50 and 74 in the 2006–2008 period, being able to complete a one-hour English-speaking survey, and living in New Jersey. Details about recruitment and retention can be found in Pruchno, Wilson-Genderson, and Cartwright (2010) and Heid et al. (2021).

People eligible for this qualitative study had completed the most recent wave of quantitative data collection (May 2020–March 2021). They lived in New Jersey cities (although New Jersey’s 564 municipalities include boroughs, cities, towns, townships, and villages, for simplicity we use the word *cities*) with the highest poverty rates and the lowest median income. Details about these cities—Atlantic City, Camden, Newark, Paterson, Salem, Trenton, Asbury Park, Bridgeton, East Orange, Elizabeth, Paulsboro, and Union City—can be found in Supplementary Material  table 1.

**Table 1.** Demographic characteristics of interviewed sample (N = 28).

Variable	Mean or percentage
Age, mean years (range)	75.0 years; (65–87 years)
Gender (female), % (n)	75.0% (21)
Race (African American), % (n)	71.0% (20)
Relationship status	
Widowed, % (n)	39.0% (11)
Married, % (n)	18.0% (5)
Divorced, % (n)	18.0% (5)
Single, never married (%)	21.0% (6)
Income, mean	$37,820
Less than $30K, % (n)	35.0% (8)
$30–$80K, % (n)	48.0% (11)
More than $80K, % (n)	13.0% (3)
Work status, % (n) retired	75.0% (21)
Years in home, mean (range)	36.0 years (15–85 years)
Household size (lives alone), % (n)	50.0% (14)
Education	
High school or less, % (n)	25.0% (7)
Some - 4 years of college, % (n)	46.0% (13)
Some graduate or more, % (n)	29.0% (8)

### Recruitment and Methods

Between June 2021 and September 2021, we sent letters in batches inviting all 90 eligible panelists to participate in a study designed to make neighborhoods friendly for older adults. We told them that, unlike the questions we had asked in the past, the questions we would ask during this interview would allow them to share details and tell stories about their experiences. We said we would ask questions about their social interactions and neighborhood resources. Nine people responded to our letter and agreed to participate. We made follow-up calls to the remaining 81 people (up to three calls each) and recruited an additional 26 participants. Others were not interested (24) or could not be located (31).

We conducted flexible semistructured interviews with 35 people. Before beginning each interview, we formally reviewed the informed consent letter approved by Rowan University’s IRB. We asked permission to audio record the interviews, which were conducted either by phone or by Zoom. The Zoom interviews were audio only. Our initial screening criteria did not include the length of time a person had lived in the neighborhood. However, as we read and discussed the interview transcripts, we realized that seven participants provided more superficial descriptions, which we attributed at least in part to their lack of a sustained relationship with their neighborhoods. This observation shifted our focus to long-term residents, and we limited our analysis to 28 participants who had lived in their homes for at least 15 years.

Our multidisciplinary team, which had expertise in gerontology, urban planning, anthropology, medicine, sociology, and qualitative research, developed the interview guide. The guide was designed to be broad enough to enable people to talk about their neighborhoods in ways that made sense to them. We piloted the interview guide before data collection and did not change it during data collection. The interview guide may be found in Supplementary Material table 2.

While our team was knowledgeable about the eight domains of AFC communities, to learn about factors most salient and relevant to our participants, the semistructured interview guide allowed for flexibility. We avoided leading questions about the AFC domains, encouraging participants to describe their neighborhoods in ways that were meaningful to them. Opening with questions to elicit these descriptions, we explored the social fabric of the neighborhood, changes in the neighborhood over time, feelings about the neighborhood, and characteristics that make neighborhoods both a good place to live and a challenging place. Finally, we asked participants about changes that could be made to improve life in their neighborhood. Interviews were conducted in a conversational tone, with probing for more detail. Although the interview guide provided a suggested order to the questions, the interview flow determined the order in which questions were asked. Probes and follow-ups were neutral and included “Can you tell me more about that?” and “Help me understand…”

Interviews lasted approximately one hour. Participants who completed the interview were mailed a $50 gift card (mentioned in the recruitment letter). Interviewers included a research assistant, the project director, and a qualitative research consultant. The project director, who has experience conducting qualitative interviews, trained the team. Interviews were recorded and transcribed verbatim; the project director reviewed each transcription for accuracy.

Most participants (23) chose to be interviewed by telephone. Modality did not create noticeable variation in the depth of the interviews or data quality. Participants were equally likely to elaborate on their responses and provide details regardless of whether interviews were conducted by phone or Zoom. Demographic data were collected at the end of each interview using standard questions (e.g., “How old are you?” and “How long have you lived at your current home?”).

### Analytic Approach

Our team met biweekly as data were being collected to discuss emerging themes. Given our interest in both the salience of the domains of the AFC framework for participants and the purposeful sampling of a theoretically unique group of long-term residents of low-income communities, our analytic approach included both deductive and inductive analysis. We conducted the initial coding round using a deductive approach (Hsieh and Shannon 2005; Elo and Kyngäs 2008), identifying instances where participants discussed AFC domains and applying codes from our coding scheme to segments of transcripts. The codes for the eight AFC domains functioned as index codes, allowing for the retrieval of segments with a discussion of the domains for further inductive coding. To facilitate this index coding, two team members created units of text around moments in the interviews ranging in length from one line to several paragraphs. This strategy has been suggested by Deterding and Waters (2021) to enable a reliable and efficient application of codes across team members by bounding moments throughout the interviews. Once coding began, the team met weekly to discuss emergent codes. We added inductive codes to the scheme, and transcripts were coded again, becoming the basis for theme development.

### Coding Scheme and Procedures for Reliability

Consistent with the AFC framework, our initial index codes included **Outdoor spaces and buildings—**the public environment (e.g., landscape, built environment, safety, crime); **Housing—**private spaces (e.g., gardens, backyards; home layout, size, security features, and affordability); **Respect and social inclusion—**accounts of older people having key roles in their neighborhood, being included socially, and the boundaries of social relationships; **Social participation—**opportunities and barriers for leisure, social, cultural, and spiritual activities; **Civic participation and employment—**opportunities to contribute to neighborhoods (e.g., paid or voluntary work, political roles, advisory councils, boards, and organizations); **Transportation—**options for using public transportation (e.g., buses, trains); **Community and health care—**the availability and range of local, municipal, state, and federal health and community support; and **Communication and information—**how communities disseminate information to older people (e.g., broadcast media, local television broadcasts, printed information, word-of-mouth, social media).

In the second coding round, based on emergent patterns in the data, codes included **Mechanisms of coping/adaptive strategies** (ways participants navigated less-than-ideal conditions), **Family proximity (**comments about a relative living nearby), **Intentions to age in place (**expressions of a desire to stay, costs associated with moving, and desire to move), and **Neighborhood change** (references to the neighborhood in years past and comparisons between what the neighborhood was like now and in the past). These emergent codes captured how participants understood the AFC domains and helped us learn how the domains related to one another. Finally, as part of each interview, participants were asked, “What would you do now to improve your life in this neighborhood?” Responses were coded as **Strengthening neighborhoods** and focused on the eight AFC domains.

First, we chose a subset of three transcripts that all team members coded and discussed to ensure that we shared the same understanding of the codes and were applying them in the same way. Next, two coders from the interdisciplinary team coded each of the remaining transcripts independently and then met to debrief and compare coding. We maximized our team’s varied disciplinary backgrounds by pairing coders with different orientations. We typically resolved discrepancies by including codes suggested by each coder and discussing as a team. This enriched our analysis by provoking additional consideration of the boundaries between different AFC domains and emerging themes. We wrote and discussed memos as a group to aid in identifying themes and guide the findings. We returned to the literature and leveraged Rowles’s (1983) concepts of insideness as a theoretical lens for understanding the themes and relationships among the AFC domains.

### Findings

Table 1 provides details about the demographics of participants. The sample included 21 women and 7 men; 20 were Black, 6 were White, and 2 were Hispanic. Participants ranged in age from 65 to 87. While the cities where participants lived were disadvantaged, the socioeconomic resources of participants were diverse. Participants had lived in their homes for an average of 36 years; several had lived in the same houses their entire lives. In reporting findings, we use pseudonyms, indicating city of residence and age in parentheses. We did not find differences across age, race/ethnicity, gender, or city.

We identified crosscutting themes in participants’ accounts. Issues regarding safety dominated the interviews and linked the domains. Participants explained how feelings of safety were enabled or constrained by outdoor spaces and homes that were or were not physically safe. Crime-related concerns, such as fear of being outside at night and being on guard for strangers in the neighborhood, linked comments about outdoor spaces, housing, communication and information, and civic participation. Participants said that feelings of safety were enhanced by respect and social inclusion, as they described social networks that included neighbors and family. Similarly, the presence of community officials made people feel safe. Another crosscutting theme linking the domains was a sense of agency. Participants described strategies they embraced for remedying problems with outdoor spaces, housing, communication and information, civic participation, transportation, and social participation.

Below, we address each domain in order of relevance to the participants, highlighting the emerging crosscutting themes and connecting our findings with Rowles’s (1983) concept of insideness. We identify changes participants said would strengthen their neighborhoods within each AFC domain.

### Outdoor Spaces and Buildings

Participants were satisfied with many of the outdoor spaces in their neighborhoods. Greenspaces, including parks, walkable sidewalks or trails, and tree-rich spaces, were places for exercise, social interaction, and aesthetic satisfaction. Joe (Paterson, 78) said, “My neighborhood is connected to the park. I walk three miles every morning.” Ellice (Newark, 67) participated in the farmer’s market, concerts, and dancing at her local park. Lila (Vineland, 73) said, “It’s very green, it’s very lush, it’s a very nice neighborhood.” Participants enjoyed living in neighborhoods with amenities. For example, Renee (Camden, 78) said, “I’m near the local hospital. Schools are within walking distance. I can walk to my church. It’s about two blocks away from me. There is a community center. It’s about a block over.” Art (Asbury Park, 70) lauded his neighborhood for its proximity to restaurants, shops, eateries, a little movie theater, ice cream, and pizza. Participants suggested how more greenspaces would benefit people of all ages.

Some described outdoor spaces that had deteriorated over time. Robert (Paterson, 87) had lived in his home since 1935 and recalled growing up there: “At one time, the local park had live deer in a pen. There were fountains and rolling hills that you could sleigh ride on in the wintertime. They’re gone. Now there are two big baseball fields down there. … The area has changed quite a bit today. There is loud music, and the park is quite full on a weekend. It’s not the same park it was.” Alma (Vineland, 70) missed a “big green area that I loved” that was gone.

Improving safety was a primary concern participants suggested would better their neighborhoods, and it was often mentioned regarding outdoor spaces. Ellice (Newark, 67) said, “The city needs to fix the sidewalks and crosswalks so they are safe for people with mobility limitations.” A few had specific strategies for improving safety that were built on years past. Olive (Newark, 87) said, “I would like to see policemen walking the street again.” Tamra (Patterson, 85) talked about the importance of someone in City Hall “who knows what to do and makes sure that stuff is done the way it should be done.” She recalled a previous mayor who “whenever stuff would happen, he would be there before the firemen got there. He would get there before the police. They knew that, so they’d go ahead and do their jobs. When you don’t have somebody watching, they don’t do what they’re supposed to do.”

Some said feelings of safety in their neighborhood had improved over time. Winston (Newark, 70) was a lifelong resident of what he described as a drug-infested city. He said, “In the early years, the city council people couldn’t do too much about it. The police didn't care. But, now you’re getting these young educated police officers that care and want to do a good job, so they’re cleaning up pretty good now.” Gertie (Trenton, 66) said, “When we first moved here, it was bad. We had people selling drugs next door and there was a shooting. Now it's not too bad. We got good neighbors. I mean they do a little drinking and partying but not half as bad what it was.” Kevin (Newark, 73) said his neighborhood was becoming gentrified. He appreciated the diversity of the neighborhood, adding that the new people are “good people, they’re nice people, they make good neighbors. I appreciate the fact that they’re all doing renovations to the homes and keeping the neighborhood from declining.”

Participants described their own ability to take action when problems arose with outdoor spaces. Erma (East Orange, 71) was an elected district leader in her community, and when a street curb needed to be cut to accommodate a wheelchair, she “took that to my city council person, and we were able to get that done.” Others described preventive efforts to maintain their neighborhoods’ outdoor spaces, which helped make them safe places. Lorraine (Camden, 69) volunteered in community programs that kept people from smoking in the park and alcohol from teenagers. She coordinated a place where people could safely drop off drugs and medicines. Ellice (Newark, 67) helped maintain parks and planned educational programming. Lorraine (Camden, 69) described an “adopt a lot” program in her community where homeowners can adopt a vacant lot next door. She said, “you cut the grass, pick up the trash, just like it was yours. When they wanna sell it, they have to come to you first and ask your permission to be able to sell it.” Some suggested turning abandoned lots into neighborhood parks and efforts to clean up the neighborhood by reducing litter and cutting weeds.

### Housing

For the most part, participants were content with their houses’ size, layout, and affordability, especially when they considered alternative places to live. Carrie (Paulsboro, 83) said, “[My ranch home] is just my size, it’s not too big and it’s not too small.” Others said that although their home was too big, they liked having the extra space. Still others said they could not afford to move. For example, Olive (Newark, 87) said, “My old house is paid. All I have to do is pay tax.” Vanessa (Patterson, 80) wanted an affordable place she could turn to for help with household chores.

Although most participants did not suffer from mobility limitations, some thought ahead to the day when they could not navigate steps. Many talked about the importance of one-story living. Others talked about living in a home with space for a caregiver. Vanessa (Patterson, 80) said, “I would like to have two bedrooms in case I can’t move or something instead of going to a nursing home. I will have somebody live with me. One bedroom will be for somebody to stay here. Free room and board and take care of me.”

While most participants could afford the homes they lived in, several worried about the cost of maintaining their homes. Rand (Newark, 68), who was living in a house that was too big and expensive for him, said the best thing to do was “sell the house to somebody who would come in and give it the care and love that it needs.” Many participants mentioned New Jersey’s notoriously high property taxes and suggested tax breaks for older people. Other financial assistance, such as free service on oil tanks and reduced fuel costs, was suggested.

Participants described a range of home modifications they made that helped them feel safe. Rand (Newark, 68) said, “I’ve got steel doors with double deadbolts. My brother and I put a six-foot cyclone fence going around for privacy. I put floodlights up around the house. It buys peace of mind. Every house on this block has been broken into at least twice except this house, and we've been here since 1952.” Others talked about their home as a safe haven—a contained space over which they maintained a sense of physical and psychological control and safety. Renee (Camden, 78) said, “Every once in a while, there’s a caravan of police around. But I know to stay in and shut my door. Once I get through the door, it’s locked and I’m in.” Janine (Newark, 72) said, “I feel safe in my house.”

### Respect and Social Inclusion

Many participants commented on respect and social inclusion in their neighborhoods. Older people played key roles in their neighborhoods. One participant said she was known as the “church lady”; another said neighbors knew her as “grandma.” Janine (Newark, 72) said people in the neighborhood respected her because “I’ve watched them grow up. They still have that level of respect for me that they had when they were kids and I was telling them to ‘take that damn pick out your head, you're not picking your hair right now, pull your pants up.’ I’m still known as Jimmie’s mother, Raven’s mother, Samuel’s mother. And I still get that respect.”

While participants described knowing about crime, they said that respect and social inclusion helped them feel more secure, as they looked out for themselves and their neighbors. Some said that although their neighborhoods were not safe, they felt safe because they knew people in the neighborhood and because they were known to people in the neighborhood. Maxine (Newark, 75) said while a stranger in the neighborhood might be in danger, no one in the neighborhood would harm her. Maxine insisted on walking a visiting nurse to her car, saying, “She didn’t know the young men, but I make sure the young men ain’t gonna do nothin’ to her and to nobody else. I knew the young men; they were raised on the block.” Winston (Newark, 70) said, “I feel pretty safe, but I keep my head on a swivel.” Always on guard, a role that made her feel valued, Lorraine (Camden, 69) said she was the neighborhood watch person. “I’m the nosey neighbor. I tell everybody, “If you goin’ do something you have no business, you better do it inside with your windows closed cause I’m the one that’ll see it!”

Long-term relationships with neighbors helped foster a sense of safety. Many participants said that family members lived in the neighborhood—either in the same house, in a connected duplex, on the same block, or around the block. Rand (Newark, 68) lived in the home he was raised in along with his older sister. Erma (East Orange, 71) lived in the downstairs unit of a duplex; her daughter and granddaughter rented the unit upstairs from her. She explained that many of her neighbors were related to one another. She said, “After my neighbor across the street moved in, the neighbor’s brother moved in down the street. Another neighbor’s sister bought the house down the street. It’s like a bunch of families living together.”

Most participants enjoyed camaraderie and engaged in reciprocal exchanges with neighbors. They looked after homes and alerted one another when they thought trouble was brewing. Ellice (Newark, 67) said she was a “connector,” active in her tenant association, where she invited elected officials and community organizations to talk to tenants. Snowstorms generally brought offers of shoveling help from younger neighbors. Janey (Trenton, 82) described a network of neighbors she could count on to take her places and help her when she needed it. She even had a hairdresser who came to her house and did her hair. When asked about improvements to their neighborhoods, some suggested that communities should do a better job of providing help to older people who live alone.

### Communication and Information

Participants said there were myriad ways to learn about what was happening in their communities. Evelyn (East Orange, 73) said, “The county calls with information every Sunday. They’re reaching out and they’re sending out communications what’s going on.” Ellice (Newark, 67), an affordable housing community resident, noted that “someone from the city or a community-based organization would often come in and talk to us about whatever was going on. We have a close relationship with our elected officials.” Others, like Bea (Elizabeth, 83), were active in staying informed via multiple sources, including social media. “They have a senior center that has a newsletter that comes out once a month to keep you abreast on what’s going on. I check on Facebook because that is how I hear about a new store, a new restaurant that's opening and activities that are going on.” The most common sources of information were family members and neighbors. During the height of the pandemic, Maria (Union City, 64) said the city communicated via landlines. Because she did not have a landline, she found out what was happening by talking to neighbors.

Participants acknowledged problems in their city but explained their neighborhood or block was safe, and at times, this perception was connected to communication and information. Some participants said the media perpetuated stereotypes that exacerbated race and residential inequality in their communities. They said their neighborhoods were no more dangerous than other neighborhoods, but that stereotypes about low-income cities amplified the perception of danger. Janine (Newark, 72) said, “There's nothing wrong with my city. … [T]here’s no more going on in Morristown than there is in Orange, wherever, Toms River. The same crap. But see—ours is broadcast. Theirs is on the down low.” Robin (Paterson, 74) said, “We don’t have a whole lotta activities around like you have in other neighborhoods where there’s people standing on the corner selling drugs and stuff. It’s not here. [But it’s] in walking distance.” Janey (Trenton, 82) said, “Our street is very good. The next street over, and the other one, forget it. It’s always something going on there.”

Safety and agency were salient when it came to modes of communication. Erma (East Orange, 71) wanted better protection for older people against scammers. Ellice (Newark, 67) defined “valuable senior activities” she’d like to see offered as those “[w]here you’re teaching seniors how to be technology-smart. Teach seniors not just how to use a cellphone to make a phone call, but (about) all the other apps. How to do Duo, how to do a Zoom meeting.”

### Social Participation

Participants said there were many opportunities in their neighborhoods for social participation. Formal activities such as “National Night Out” provided opportunities to meet neighbors, local law enforcement, and political leaders. Others described informal gatherings. For example, Lorraine (Camden, 69) said her neighborhood was a close-knit, multigenerational community that often gathered informally in a vacant lot between houses. “It’s just an empty lot. But when we have our cookouts, we put our grills out there. We just have a good time.” Participants who identified gaps in opportunities for social participation suggested that offering activities at alternate times of day and ensuring that the space in which these activities take place is handicap accessible would increase opportunities for older people to participate.

### Civic Participation and Employment

As previously described, many participants discussed the active roles they play in their neighborhoods, through civic participation and employment. Robin (Paterson, 74) worked part-time as a school bus aide. Erma (East Orange, 71) was an elected district leader in her community. Alma (Vineland, 70) volunteered for the American Cancer Society. Several people were active in neighborhood block clubs and tenant associations. Even though Art (Asbury Park, 70) worked full-time and commuted over an hour each day by train, he considered running for school board membership; “I’m an educator by profession.”

### Transportation

Public transportation was valued by people living in neighborhoods with access to it and wanted by people without access. Public buses empowered people to get to places they otherwise could not go because of physical limitations. Gertie (Trenton, 66) said, “I can’t walk real far too much, but I usually take a bus. Buses are close. You know, I can get on the bus and go where I gotta go.” Access to public transportation, however, required learning. For some, avoiding learning new transportation routes was a reason to age in place. Ellice (Newark, 67) said, “If I moved, it would be a whole new learning curve, and do I really want to do that?”

### Community and Health Care

Community and health care was often provided at the local senior centers. Janey (Trenton, 82) said, “They deliver food to me. And then once in a while they call me to see how I’m doing.” Maxine (Newark, 75) said she went to the local senior clubhouse that offered dancing, exercise, Zumba, day trips, and various social activities. “We’d go down there and we’d have lots of fun. You get to know new people, something to do and enjoy yourself and to get out, so to be sociable as well as to get in exercise and things like that.” Some, like Lorraine (Camden, 69), talked about the integral role of the postal carriers and trash collectors in the neighborhood. She said, “The trash people go above and beyond their job to help older people. If my trash is not outside on the curb, they will come in the yard, and get it, dump it, and put my cans back in.” Of the postal carrier, she said, “So I don’t have to walk down the steps, my mailman brings the mail to me.” Some of the Camden participants told us that one of the neighborhood pharmacies had a program where they gave out free books and helped with bills. Others in that city said a corner store and bakery provided free food to homeless people. Several participants said that increasing proximity and access to community and health care would improve their neighborhood for older people. Bea (Vineland, 83) said, “Most of the senior services the city has are not close to this area.”

## Discussion

This analysis adds new information by examining the applicability of the AFC domains in low-income cities from the perspective of long-term residents. Although participants provided their own descriptions of their neighborhoods in response to our prompts, we were able to index their descriptions with analytic codes corresponding to the eight domains of the AFC framework. This suggests that the AFC domains are a useful heuristic tool for understanding the experiences of older adults living in low-income cities.

However, our analysis moved beyond the AFC domains as themes of safety and agency emerged throughout participants’ accounts. Safety was related to several domains: outdoor spaces, housing, respect and social inclusion, communication and information, and civic participation. Participants experienced agency, or a sense of control, in several domains, including outdoor spaces, housing, communication and information, social participation, civic participation, and transportation. Some participants described themselves as actively working to improve the safety of their neighborhoods. Our findings suggest the need for additional research on safety and agency as they relate to aspects of neighborhood environments. Our findings also lend support to proposed refinement of the original AFC domains to emphasize informal social support and safety (Plouffe, Kalache, and Voelcker 2016).

Although our interview questions encouraged people to talk about their neighborhoods in the present, many reflected on their accumulated experience there. Participants had lived in their neighborhoods for years, many for decades, and some for their entire lives. Consistent with Rowles’s (1983) conceptualizations of *physical insideness*, our participants were intimately familiar with their neighborhoods’ outdoor spaces, housing, and transportation. Yet the relationships older people have with their physical environments are complex, particularly regarding safety and agency. While participants valued the amenities and public transportation proximate to their neighborhoods and the agency these afforded, they were aware of their neighborhoods’ dangers, including drugs, crime, and vacant homes. Many appreciated the size, space, and layout of the homes in which they lived. They valued the spaces they owned outside the walls of their homes—their gardens, porches, and backyards. Yet, the affordability of these homes was of concern. While many participants lived in homes already paid off, they expressed concern about New Jersey’s high property taxes and the costs of running and maintaining their older homes. And yet, many were able to remain in the neighborhood by either hiring help or receiving informal help from neighbors.

Consistent with Rowles’s (1983) notion of *social insideness*, participants were well integrated with their neighborhoods’ social fabric. They discussed their daily lives in their neighborhoods in ways that linked to the AFC domains of respect and social inclusion, social participation, civic participation and employment, and communication and information. For many, this social insideness enabled them to navigate the challenges posed by the physical aspects of their neighborhoods over which they lacked control. Respect and social inclusion was an AFC domain that helped explain why participants felt safe in their neighborhoods despite dangers. Whether neighbors viewed them as “church lady,” “grandma,” or someone’s mother, these roles made participants feel known, which in turn helped them feel safe. Like the “natural neighborhood networks” described by Gardner (2011), we found that the vibrant informal social networks in their neighborhoods, including friendly conversations, help shoveling snow or securing groceries, and watching one another’s home, made participants feel that they were not alone. Living in neighborhoods that included family members—children, siblings, and grandchildren—promoted respect and social inclusion and encouraged social participation. It enabled participants to get where they needed to go and made them feel safe. Our finding aligns with others suggesting that social capital and cohesion are pathways to age friendliness in diverse urban neighborhoods (Parekh et al. 2018).

Our findings build on Heid et al. (2017), who documented the physical and socioemotional help neighbors provided to older people before, during, and after Hurricane Sandy. In that study, although participants described a unique friendliness as neighbors came together to combat adversity, participants said this friendliness was short lived, as people resumed “typical patterns of individualism” that existed before the storm. However, these new data suggest that ongoing natural helping networks are vibrant and meaningful in low-income cities. We found that although some neighbors left and new neighbors moved in over time, the respect and social inclusion characterizing a neighborhood was not contingent on how long people lived there. Long-term residents know their neighbors, and these neighbors play important roles for one another. Future studies should examine how natural neighborhood networks develop and how these connections promote interdependence among people of all ages, enabling older people to age in place.

Consistent with Rowles’s (1983)  *autobiographical insideness*, our participants had lifelong accumulations of experiences in their neighborhoods that provided them with a sense of identity. Having witnessed changes as far back as the 1960s race riots, participants watched the ethnic composition of their neighborhoods change. They watched old neighbors leave and new neighbors—often people very different from them—move in. They saw children grow up and leave the neighborhood. They saw stores go out of business and main streets close. They lived through periods of crime and safety. Some witnessed trees being cut down and parks where they played as children deteriorate. Some watched houses become vacant and vegetated. But some saw police taking a more active role in combatting crime and mayors implementing changes designed to improve their communities. Some saw people buy and renovate vacant houses, breathing new life into their neighborhoods. As insiders, our participants experienced a unique sense of purpose and connection to the neighborhood. It is possible that this insideness and the deep connections our participants had in their neighborhoods kept them from leaving the neighborhood. Like Plasencia’s (2022) finding, our study contradicts the idea that people age in place in low-income cities because they have no alternative or are “stuck in place.” While most of our participants expressed intentions to age in place while talking about what makes their neighborhood a positive or challenging place, this work is left for future studies because we did not explicitly ask about intentions to leave or to age in place.

Buffel and colleagues (Buffel, Handler, and Phillipson 2019; Lewis and Buffel 2020) wrote that “sociological studies demonstrate how the neighborhoods in which people age may prove to be hostile and challenging environments, influenced, in part, by urban change arising from globalization, urban regeneration and austerity” (Lewis and Buffel 2020, p. 1). In this light, people’s attributions about their neighborhood or block feeling safe deserve attention. This distancing from drugs and shootings, claiming they were not problems on “my block,” may have been what enabled older people to continue living in their neighborhoods. Future research examining how low-income city residents define their neighborhoods’ physical boundaries is important.

Older people played active, critical civic roles in their neighborhoods, volunteering in the churches, schools, and neighborhood associations to improve the places where they lived. Acknowledging long-term residents not as passive recipients of neighborhoods but as capable agents shaping their neighborhoods through their daily routines and intentional participation is important for the continued development of age-friendly cities.

Understanding the perspectives of older African American women living in low-income cities, a group underrepresented in research, is a strength of this study. However, there are other groups of people living in low-income cities (e.g., Asians, Latinos/as) whose stories we do not know. Moreover, findings cannot be generalized to the experiences of people living in neighborhoods we did not study. As Golant (2015) suggests, places are specific and have unique characteristics. Cities and neighborhoods are diverse and not static. We acknowledge that these specific findings may be unique to the neighborhoods in this study at the time of the study. Because we did not include people who had lived in their neighborhoods for less than 15 years, we acknowledge that findings may not pertain to people who relocated to these neighborhoods in their later years. Rather, our findings pertain to people who aged in place. However, our findings may also be transferable in their applicability to other similar contexts and their resonance with experiences of older adults elsewhere. Although all participants lived in low-income cities, not all were impoverished; half reported incomes over $50,000. Unfortunately, we had too few people to make meaningful comparisons among socioeconomic groups, yet future research focused on people with means choosing to live in impoverished cities would provide an important perspective.

The flexible, semistructured nature of the interviews is both a strength and a limitation of this study. As a strength, we learned that the AFC domains are salient even when participants are not asked about them explicitly. As a limitation, the ordering and flexibility of the semistructured interview may have affected the stories participants chose to tell and which domains were mentioned most often. Moreover, it is important to acknowledge that we relied on a convenience sample from a panel, given the difficulties of finding and recruiting long-term residents. Despite these limitations, our findings highlight the importance neighborhoods have for long-term residents living in low-income cities, suggest pathways for improving the lives of older people, and enrich theory about how older people engage with their neighborhoods.

Finally, our findings suggest how low-income cities can become more age friendly. Participants identified changes to outdoor spaces (e.g., bringing buildings up to code, ensuring there were places where residents could purchase fresh fruits and vegetables, repairing fractured sidewalks, turning abandoned lots into parks, increasing police presence, cleaning up trash and drug use sites), housing (e.g., repairs, modifications, tax incentives), and community and health care (e.g., optimizing place, variety, and times for services). Although many of these changes are costly and require city commitment, benefits exist for people of all ages. However, our study shows the importance of older people feeling respected and included in social activities. While our participants described social relationships that developed organically in their neighborhoods, interventions that encourage neighbors to work collaboratively on a problem (e.g., vacant lots, crime) can better the neighborhood’s social fabric, making it more age friendly, while being less costly for cities.

By examining the experiences of older people who have lived in low-income cities long term, our findings add to knowledge about what an age-friendly city is and find that low-income cities can be age friendly. Affordable housing, neighbors who look out for and help one another, and proximity to shopping, amenities, and public transportation are central to an older person’s ability to maintain independence and age in place. However, despite these benefits, many urban, low-income cities experience challenges resulting from decades of disinvestment that make them potentially unsafe places to grow old. In sum, the age-friendly domains provide a useful context for understanding the neighborhoods in which people age.

## Supplementary Material

nfaf027_Supplementary_Data

## Data Availability

Data, Materials, and Analytical Code Transparency: We cannot share raw data due to IRB restrictions. However, we provide examples of our coding process and the interview guide herein.
