# Supplementary material for: How the Age-Friendly Domains Apply to Low-Income Cities and Guide Improvements: Perspectives of Long-Term Residents in New Jersey
Source: Public Opin Q. 2025 Aug 7;89(SI):517–38. doi: 10.1093/poq/nfaf027 (PMC12411912; doi:10.1093/poq/nfaf027)
Supplement: nfaf027_Supplementary_Data [file nfaf027_supplementary_data.pdf]

Supplementary Material

How the Age-Friendly Domains Apply to Low-income Cities and Guide Improvements: Perspectives of Long-Term Residents in New Jersey

Amanda Jones-Layman, Ph.D., School of Social Sciences, Humanities, and Education, Neumann University; joneslaa@neumann.edu

Francine P. Cartwright, B.S., New Jersey Institute for Successful Aging, Rowan-Virtua School of Osteopathic Medicine; cartwright@rowan.edu

Seran Schug, Ph.D., Department of Sociology and Anthropology, Rowan University; schug@rowan.edu

Jennifer Kitson, Ph.D., Departments of Art and Geography, Planning, & Sustainability, Rowan University; kitson@rowan.edu

Lisa Siegert, M.D., FACP, Division of Geriatric Medicine, Cooper University Health Care; Siegert-Lisa@CooperHealth.edu

Rachel Pruchno, Ph.D. New Jersey Institute for Successful Aging, Rowan-Virtua School of Osteopathic Medicine; pruchnra@rowan.edu

Supplementary Material Table 1. New Jersey Municipalities: Socioeconomic Indicators and Recruitment Status

Supplementary Material Table 2. Interview Guide

Supplementary Material Table 1. New Jersey Municipalities: Socioeconomic Indicators and Recruitment Status

Population below poverty level		Municipality	Median household income		Number of ORANJ BOWL panelist		
(%)	NJ RANK		NJ RA NK	($)	Met eligibility criteria	Total recruited	Years at current residence≥15
42.18	1	Salem	1	24,926.00	4	0	0
37.08	2	Atlantic City	3	29,232.00	2	1	0
36.38	3	Camden	2	27,015.00	5	3	2
34.39	4	New Brunswick	16	43,783.00	2	0	0
31.19	6	Bridgeton City	8	37,804.00	5	1	0
28.72	7	Trenton	6	35,402.00	12	3	2
28.64	8	Passaic	10	40,865.00	1	0	0
27.41	9	Newark	5	35,199.00	17	9	9
26.60	10	Paterson	12	41,360.00	13	5	4
25.83	13	Asbury Park	23	47,841.00	1	1	1
24.22	16	Lakewood	34	52,148.00	1	0	0
19.65	26	Union City	28	48,992.00	2	1	1
18.03	35	Paulsboro	20	45,450.00	3	2	1
17.99	36	East Orange	24	48,072.00	7	4	3
17.63	37	Elizabeth	25	48,407.00	5	2	2
13.67	61	Vineland	42	54,476.00	10	3	3

U.S. Census Bureau (2015-2019). *Median Household Income in the Past 12 Months (In 2019 Inflation-adjusted Dollars) American Community Survey 5-year estimates.* Retrieved from https://censusreporter.org https://censusreporter.org/data/table/?table=B19013&geo_ids=04000US34,060|04000US34&primary_geo_id=04000US34;

U.S. Census Bureau (2015-2019). *Poverty Status in the Past 12 Months by Sex by Age American Community Survey 5-year estimates.* Retrieved from <https://censusreporter.org https://censusreporter.org/data/table/?table=B17001&geo_ids=04000US34,160|04000US34&primary_geo_id=04000US34

Information about denominators for poverty and income: https://www.census.gov/programs-surveys/saipe/guidance/model-input-data/denominators.html

Supplementary Material Table 2. Interview Guide
1. Tell me about where you live.<br>2. Tell me about what your neighborhood was like when you first moved in.<br>3. Tell me about the people whom you see and talk with.<br>4. How do you feel about living in your neighborhood?<br>5. Who provides help when (or if) you need it?<br>6. What aspects of your neighborhood make it a good place for you to live?<br>7. Which aspects of your neighborhood cause you concern or worry?<br>8. How has the pandemic affected you?<br>9. What would you do right now to improve your life in this neighborhood?
